# Metabolic characterization of alkane monooxygenases and the growth phenotypes of *Pseudomonas aeruginosa* ATCC 33988 on hydrocarbons

**DOI:** 10.1128/jb.00508-24

**Published:** 2025-03-11

**Authors:** Thusitha S. Gunasekera, Loryn L. Bowen, Jhoanna C. Alger

**Affiliations:** 1 Fuels and Energy Branch, Aerospace Systems Directorate, Air Force Research Laboratory, Wright-Patterson Air Force Base 97045 , Dayton, Ohio, USA; 2 University of Dayton Research Institute, University of Dayton 2824 , Dayton, Ohio, USA; University of Illinois Chicago, Chicago, Illinois, USA

**Keywords:** *Pseudomonas aeruginosa*, alkane degradation, gene expression, alkane monooxygenase, phenotypic heterogeneity, substrate specificity/flexibility, VBNC cells, flow cytometry

## Abstract

There is a demand and widespread interest in evaluating microbial community structures and metabolic processes in hydrocarbon environments. The current work aims to detect microbial subgroups (phenotypic subsets) and their metabolic processes, such as substrate specificity and expression of niche-associated genes. In this study, we were able to discriminate different cell types in real time from a complex sample matrix to allow the detection of live, dead, and injured cell populations in jet fuels. We found that the expression of *alkB1* and *alkB2* genes is induced in a growth-dependent manner and *alkB2* induction started before *alkB1*. This indicates that as an early response of *Pseudomonas aeruginosa* cells’ exposure to alkanes, cells activate *alkB2* gene induction. Deletion of *alkB1* and *alkB2* genes completely inhibited *P. aeruginosa* ATCC 33988 growth in jet fuel, suggesting that two alkane monooxygenases are responsible for the degradation of alkanes and jet fuel. Interestingly, the AlkB2 has a broader (*n*-C8*-n*-C16) substrate range compared to AlkB1 (*n*-C12-*n*-C16). The data indicate that two alkane utilization pathways can coexist in *P. aeruginosa* ATCC 33988, and they are differentially expressed in response to *n*-C6*-n-*C16 alkanes found in jet fuel. This study provided additional information on the heterogeneity and phenotypic diversity within the same species after exposure to hydrocarbons. This work advances our understanding of microbial community structures and provides new insight into the alkane metabolism of *P. aeruginosa*.

**IMPORTANCE:**

Alkane degradation allows for the natural breakdown of hydrocarbons found in crude oil, which can significantly contribute to environmental remediation. The metabolic process of microbes to hydrocarbons and the expression of niche-associated genes are not well understood. *Pseudomonas aeruginosa* ATCC 33988, originally isolated from a jet fuel tank, degrades hydrocarbons effectively and outcompetes the type strain *Pseudomonas aeruginosa* PAO1. In this study, we found differential expression of *alkB1* and *alkB2* alkane monooxygenase genes and the relative importance of these genes in alkane degradation. We found different phenotypic subsets within the same genotype, which are influenced by hydrocarbon stress. Overall, the research conducted in this study significantly contributes to our knowledge about microbial processes and community structure in hydrocarbon environments.

## INTRODUCTION

Microorganisms in the environment exist as a dynamic and diverse population in complex ecosystems (1). They are exposed to a variety of environmental stresses, and these stress factors can destabilize their community structure. While some microorganisms are susceptible to environmental stresses, some are metabolically poised to respond to such challenges. Even within the same genotype, phenotypic heterogeneity has often been seen under environmental stress (2). Therefore, systematically detecting and analyzing the dynamic behavior of microbial populations without affecting their actual niche are important for microbial ecologists to understand their metabolic activity in a particular ecosystem. Microbial contamination of petroleum-derived hydrocarbons in environments, such as water and soil, triggers the colonization of hydrocarbon-degrading microorganisms at contamination sites. In the event of hydrocarbon contamination of the environment, rapid shifting of a microbial population was observed (3), and well-adapted hydrocarbon-metabolizing microorganisms dominate in the microbial consortium (4). Hydrocarbon-degrading microorganisms also produce metabolites that induce syntrophic and co-metabolic processes (5). Characterization of hydrocarbon-degrading bacteria, their metabolic flexibility, and physiological state is important in developing ideal biodegradation strategies. Microorganisms use different or preferred transformation pathways to remove environmental contaminants (6). Jet fuel is composed of a wide range of hydrocarbons, including normal paraffins, isoparaffins, cycloparaffins, and aromatics, such as alkylbenzenes, Indane, tetralins, naphthalene, and alkylnaphthalenes (7). Jet-A contains approximately 22% aromatics and 24% normal alkanes, with the balance containing isoparaffins and cycloparaffins (7). Microorganisms have evolved specific cellular mechanisms to survive in hydrocarbon-rich environments. Microbial adaptive mechanisms, such as secretion of exopolysaccharides and biofilm formation, iron uptake pathways, solvent-resistant mechanisms like efflux of toxic solvents, and stress response mechanisms, are key mechanisms that microbes utilize to survive in the fuel environment (8–14). Hydrophobic properties of microbial cell surfaces are advantageous to interact with hydrophobic compounds, allowing for a stronger adhesion and binding to non-polar substances (15).

*Pseudomonas aeruginosa* ATCC 33988, originally isolated from a jet fuel tank, degrades hydrocarbons effectively and outcompetes the type strain *Pseudomonas aeruginosa* PAO1 (8, 9). The *Pseudomonas aeruginosa* ATCC 33988 genome encodes two alkane monooxygenases (16), but their relative importance in alkane degradation is not known. Transcriptomic studies confirmed that two alkane monooxygenases are upregulated in the presence of jet fuel, with *alkB2* being upregulated higher than *alkB1* (8, 9). Recent elegant structural studies on AlkB proteins have revealed a possible model for substrate binding that dictates alkane monooxygenase function and specificity (17–19). Different AlkB proteins have unique specificity to the chain length of alkanes that they oxidize. Recent studies on AlkB of *Fontimonas thermophilia* demonstrate that the AlkB forms a hydrophobic substrate channel that accommodates the alkane molecules with the support of multiple leucine residues, orienting the terminal C-H bond toward a diiron active site. The diiron active site is essential for activating C-H bonds, and they are coordinated by nine histidine residues (20–22), which are well conserved in different AlkBs, including *Pseudomonas aeruginosa* PAO1 and ATCC 33988 strains (9). Knocking out individual *alkB* genes helps to understand the relative importance of each *alkB* gene on hydrocarbon degradation, and creating a double mutant allows studying the combined effects of losing both genes at once. Therefore, our study is focused on the relative importance and activity of these two *alkB* genes on hydrocarbon degradation and their ecological role, related to their hydrocarbon-rich environment. Differential expression of these genes in the presence of different hydrocarbons and their induction depending on the length of the carbon chain allows the ability to degrade different hydrocarbons. Once alkanes are hydroxylated by these two alkane monooxygenases, the downstream molecular pathways that are used to oxidize alcohols to fatty acids and the beta oxidation pathway by which fatty acids are broken down into acetyl-CoA are well understood. The rate-limiting enzymatic reaction in the metabolic pathway involves two alkane monooxygenases, and currently, their relative expression and substrate specificity have not been elucidated.

Although the CrgA regulator was identified as a negative regulator for the *alkB2* gene of *P. aeruginosa* (23), the complete mechanism of *P. aeruginosa* regulation of *alkB* genes is unclear. Since *alkB1* and *alkB2* genes have specific substrates and expression dynamics, there should be a unique mechanism that regulates *alkB* genes in *P. aeruginosa*, most likely involving different regulatory proteins or signaling pathways that respond to alkanes, particularly different chain length alkanes, which are their primary substrates in jet fuel. In contrast, *P. putida* regulation of *alk* genes is well understood and is primarily controlled by the transcriptional regulator protein AlkS, which is activated by the presence of alkanes. AlkS is regulated by other global regulators, including Crc and Hfq, where Crc acts as a primary regulator and Hfq acts as an RNA-binding protein to facilitate Crc interaction with target mRNAs, effectively controlling the expression of AlkS at the post-transcriptional level, particularly in the context of carbon catabolite repression (24, 25).

Hydrocarbons are hydrophobic due to their nonpolar nature, making it difficult to use directly in analytical equipment to assess microbial content. The rapid and quantitative detection of microbial communities in hydrocarbon-contaminated environments or microbially contaminated fuel tanks is hampered by the lack of sensitive methods to detect microorganisms. Having a rapid and sensitive detection of phenotypic subsets, such as viable, non-viable, and viable but nonculturable (VBNC) cells, can assist in understanding microbial survival strategies in the environment in response to hydrocarbons. Currently, several methods are used for the detection and enumeration of microorganisms in hydrocarbon-contaminated sites or jet fuel tanks, including culture-based methods and quantitative PCR (qPCR)-based assays (26). Culture techniques are the most common, but major disadvantages include the time needed to produce results and failure to enumerate VBNC organisms (27–29). Environmental factors and stresses can affect cell division and cause microorganisms to become non-culturable (27–29). These VBNC cells often show less metabolic activity (29) yet can express genes (27) and are able to resuscitate and reestablish full metabolic activity when conditions are favorable (29). The VBNC state is accepted as a bacterial mechanism of survival when confronted with external stresses. Therefore, VBNC cells are significantly underestimated in the plate count method if microbes are enumerated purely based on cell division and reproduction.

Quantitative PCR-based assays are rapid, but they fail to distinguish microbial DNA from live vs dead cells and significantly overestimate functional microbial populations. However, recent attempts have been made to exclude non-viable cells’ DNA from samples prior to quantitation by PCR (30, 31). Biochemical and immunological-based assays are fast, yet they lack absolute specificity and cannot capture the depth of heterogeneity within microbial populations. These limitations are particularly magnified when dealing with dynamic and diverse populations containing active functional cells compared to dead and “injured” cells. It is, therefore, doubtful whether these approaches provide a true picture of microbial activity in hydrocarbon-contaminated sites or fuel tanks. Thus, viability staining and sub-grouping live/dead/injured cell populations can help users predict and apply control measures effectively.

Flow cytometry (FCM) can detect and differentiate cells into different cell types and determine their physiological state using functional fluorescent stains (32, 33). Recent reviews have discussed the scope of FCM and applications in various areas of the field (34–36). Advances in instrumentation and the development of a vast array of functional fluorescent stains have enabled the collection of a wide range of cellular characteristics in real time. Although FCM is primarily used for the analysis of human cell types, such as immune cells targeting cell-surface markers, it has made a significant advancement to other areas in biology, including microbiology. This technology has been successfully used to differentiate microorganisms from complex environments, such as milk (37–39), beer (40), wine (41), water (42, 43), urine (44), probiotics (45), pathogenic bacteria (46), and microorganisms in biological fluids (47). FCM has recently been used to quantitate live/dead and viable but nonculturable cells when microbes are exposed to novel antimicrobial compounds (48, 49). Green fluorescent protein (GFP) is a sensitive and ideal reporter used in conjunction with FCM to study gene expression, particularly as a quantitative reporter to assess different inducible genes (50). Here, we evaluate microbial growth in fuel, fuel/water interface, and water phase and studied their dynamic and diverse populations after exposure to hydrocarbon stress. The growth and hydrocarbon degradation rates of *P. aeruginosa* ATCC 33988 wild type and *alkB1* and *alkB2* mutant strains were characterized by growth assays in different alkanes and gas chromatography-mass spectrometry (GC-MS) analyses. Furthermore, we show a temporal pattern of expression of alkane monooxygenase genes (*alkB* genes) in *P. aeruginosa* ATCC 33988.

FCM is able to recover and discriminate different cell types in real time from the sample matrix, allowing differentiation of live, dead, and injured cell populations simultaneously and is used as a tool to understand microbial processes, such as gene expression of niche-associated genes. Here, we show the temporal pattern of expression of alkane monooxygenase genes (*alkB* genes) in *P. aeruginosa* ATCC 33988 using *alkB* mutants. Understanding the contribution of different alkane monooxygenase activity, their transcriptional responses, and substrate specificity/flexibility are crucial for their adaptability to hydrocarbon-rich environmental niches and understanding biodegradation potential for developing improved bacterial systems for biotechnological and environmental applications. Overall, this study advances our understanding of microbial community structures and microbial processes in hydrocarbon environments.

## MATERIALS AND METHODS

### Flow cytometry and microbial sample preparation

The Attune NxT (Invitrogen), an acoustic-assisted flow cytometer equipped with a blue (488 nm)/yellow (561 nm) laser with different fluorescent channels, was used in this study. Common fuel microorganisms, such as *Pseudomonas aeruginosa* (16), *Pseudomonas putida*, *Pseudomonas stutzeri* (51), *Gordonia sihwensis* (52), *Nocardioides luteus* (53), and *Yarrowia lipolytica* were inoculated into fuel individually. In order to distinguish bacteria from yeast in flow cytometry scatterplots, either 100% yeast cells (*Yarrowia lipolytica*) or 100% bacteria (*P. putida*) were inoculated, and different regions (sub-populations) were gated on scatterplots to make sure bacteria and yeast could be visualized and identified on flow cytometry scatterplots in a mixed population. To validate if bacteria and yeast could be detected simultaneously on the scatterplots in a mixed population, approximately 5% yeast culture (cells/mL) was spiked into a bacterial culture. Bacteria were grown in Luria-Bertani (LB) medium overnight at 28°C. Unless otherwise stated, M9 minimal medium was used for bacteria growth, and Yeast Nitrogen Base minimal medium was used for fungal growth. Cells were washed twice using phosphate-buffered saline (PBS), resuspended in minimal media (M9), and inoculated at an optical density at 600 nm (OD_600_) of 0.001 into 10 mL of minimal medium overlaid with 10 mL of Jet A fuel in 50 mL polypropylene tubes. Sample tubes were incubated at 28°C and shaken at 200 rpm, and samples were taken from the water phase, fuel/water interphase, and fuel phase periodically. The bacteria in the contaminated water phase were directly sampled, but bacteria at the fuel phase/interphase were extracted to the liquid phase using the liquid/liquid extraction method as described previously (26). The liquid was then stained using fluorescent dyes before analyzing using the flow cytometer.

### Live/dead staining

SYTO 9 is a cell-permeable blue-excited (488 nm), green-fluorescent nucleic acid dye that can enter cells regardless of their membrane integrity, intercalate with nucleic acid stains, and emit green fluorescence, while propidium iodide (PI) can only enter compromised bacterial membranes, making it an excellent probe for identifying dead cells (54). This principle has been proven widely in many systems and environments where microbes interact with different environmental stresses and molecules, although PI staining can underestimate the viability of adherent bacterial cells (55). On an Attune flow cytometer, the PI was excited by 488 or 561 nm and was detected in a 610/20 bandpass. This intercalating DNA dye exhibits a 20–30-fold increase in fluorescence when binding to DNA. SYTO 9 is a blue-excited (485 nm excitation maximum), green fluorescent (498 nm DNA, 501 nm RNA emission maxima) nucleic acid stain, while PI has an excitation maximum of ~535 nm and an emission maximum of ~615 nm. For live/dead cell determination, cells were stained with SYTO 9 and propidium iodide at a final concentration of 2.5 µM and incubated in the dark for at least 20 min before analyzing through the flow cytometer. The threshold was set at green fluorescent detectors and yellow fluorescent detectors to remove background fluorescent particles. The live and dead cells were gated on dot plots and analyzed separately. Subsamples were diluted and plated on Tryptic Soy Agar (TSA) media for bacteria, and viable colonies/mL of the samples were estimated. For the enumeration of colony-forming units (CFUs) for yeast, samples were diluted and plated on Yeast Peptone Dextrose media. For the extraction of microorganisms from the fuel phase, an equal volume of PBS (pH 7.4) was mixed with the fuel and vortexed for 1 min. The microorganisms contained in the PBS aqueous phase were then stained with appropriate fluorescent dyes before analysis. *Byssochlamys* isolate BYSS01 (56) cultures were grown on Potato Dextrose Agar medium until sporulation. Ten milliliters of PBS solution was added to the culture plates, and the fungal spores were harvested. The mixtures containing mycelia and spores were filtered through 10 µm mini strainers (pluriSelect, USA), and the resulting spore suspension was concentrated by centrifugation at 10,000 rpm for 5 min. Spore counts/mL were estimated using a hemocytometer, and different spore concentrations were added to fuel samples (10 mL), with the aqueous phase containing 5 mL PBS. Tubes were vortexed thoroughly and left several minutes before collecting samples from the fuel/water interface for analysis. Fungal spores were stained with 2.5 µM of SYTO 9 and incubated for 20 min before analyzing them through the flow cytometer.

### Effect of fuel additives on microorganisms and detecting viable non-culturable cells using flow cytometry

Diethylene glycol monomethyl ether (DIEGME), an additive added to aviation fuels that prevents the formation of ice in a fuel system, has proven antimicrobial properties (57). DIEGME was added to fuel/buffer in different concentrations, ranging from 0%, 0.1%, 0.5%, 1%, 2.5%, and 5.0%, and mixed well. *Pseudomonas aeruginosa* ATCC 33988 cells were added at 6 × 10^5^ cells/mL, and tubes were incubated at 28°C and shaken at 250 rpm. Samples were taken periodically and tested for viability using the live/dead assay and culture plate method.

### Alkane monooxygenase genes’ (*alkB1* and *alkB2*) expression using flow cytometry

*P. aeruginosa* ATCC 33988 has functional *alkB1* and *alkB2* genes, which are essential for survival in alkane-rich substrates such as jet fuel. The objective of this experiment was to evaluate the induction timeline for *alkB1* and *alkB2* genes using flow cytometry. In our previous study, the *alkB1* and *alkB2* promoters of *P. aeruginosa* were fused to green fluorescent protein, cloned into the pHERD20T plasmid (58), and transformed into *P. aeruginosa* ATCC 33988 strains. The strains carrying these plasmids were used for this study along with *P. aeruginosa* carrying a pHERD20T empty plasmid. The cells were grown in 0.2% glycerol and Jet A, and the GFP expression was evaluated over time using flow cytometry. Cultures were inoculated at an optical density (OD) of 0.001 in 10 mL minimal medium with 500 µg/mL carbenicillin and grown at 28°C with shaking at 250 rpm. GFP expression was measured periodically using flow cytometry.

### Generation of *alkB1 and alkB2* mutants and growth analysis

The deletion mutants of alkane monooxygenase 1 and alkane monooxygenase 2 and the double mutant were created using homologous recombination allelic-exchange method described previously (59–61). The upstream and downstream regions of the position of *alkB1* and *alkB2* mutants to be introduced were PCR amplified and cloned to a suitable suicide vector and transformed into *P. aeruginosa* ATCC 33988 strain to obtain double cross-over events. While *Escherichia coli* strain DH5α was used as a host strain for plasmid construction and propagation, *Escherichia coli* S17-1 λpir was used as a donor strain, which has conjugative machinery to integrate DNA to the target organism, *P. aeruginosa* ATCC 33988. *Saccharomyces cerevisiae* strain InvSc1 (Invitrogen Corporation) was used for the *in vivo* homologous recombination to modify plasmids. The *P. aeruginosa* ATCC 33988, *Escherichia coli* DH5α, and S17-1 λpir strains were routinely cultured in LB medium, while *S. cerevisiae* strain InvSc1 (Invitrogen) was grown with yeast extract-peptone-dextrose (1% Bacto yeast extract, 2% Bacto peptone, and 2% dextrose). The deletion of *alkB1* and *alkB2* genes was confirmed using primers described in Table 2. Deletion in *alkB1* removed approximately 1,038 base pairs, resulting in a deletion of 355 of the 382 amino acids from the full-length AlkB1 protein. Similarly, the deletion of *alkB2* removed 354 of 377 amino acids from the full-length AlkB2 protein. The Δ*alkB1* Δ*alkB2* double mutant was constructed by the deletion of the *alkB2* gene in the Δ*alkB1* mutant.

The primers that are used to amplify gene fragments are listed in Table 1. We confirmed the intended mutations in the gene of interest by PCR amplification and subsequent Sanger sequencing. *Pseudomonas aeruginosa* ATCC 33988 and *alkB* mutants were grown in LB medium overnight at 28°C. Cells were washed twice using 1× phosphate-buffered saline and inoculated into 20 mL of 1× M9 minimal medium overlaid with 5 mL of Jet A fuel in 50-mL polypropylene tubes. The initial inoculum was set at an optical density at 600 nm (OD_600_) of 0.03. Samples were incubated at 28°C and shaken at 200 rpm, and the OD_600_ data were collected periodically using a spectrophotometer.

**TABLE 1:** Primers used for *alkB1* and *alkB2* gene mutation

*alkB1_frag1F*	GACGTTGTAAAACGACGGCCAGTGCCAAGCTTGCATGCCTCTTGAAGCGTTCGCTTTCGGCGATGAAG
*alkB1_frag1R*	CTGTTCGGCCGTCAACTGGAACTCTCTAGTTAGCTAGCAGAGCCAATAGGCGTACTTCTTGATG
*alkB1_frag2F*	CATCAAGAAGTACGCCTATTGGCTCTGCTAGCTAACTAGAGAGTTCCAGTTGACGGCCGAACAG
*alkB1_frag2R*	GCTCGTATGTTGTGTGGAATTGTGAGCGGATAACAATTTCGATGGATTGGGAGGTGTCGTGGAG
*alkB2_Frag1F*	GACGTTGTAAAACGACGGCCAGTGCCAAGCTTGCATGCCTTGGTCTCGGCGTTGTCGAAGATCAG
*alkB2_Frag1R*	GTAAGCTGGTATTCCTCGCCGGCATCTAGTTAGCTAGGATCAGCCAGATCCAGTAGCCGTACTT
*alkB2_Frag2F*	AAGTACGGCTACTGGATCTGGCTGAT**C**CTAGCTAACTAGATGCCGGCGAGGAATACCAGCTTAC
*alkB2_Frag2R*	GCTCGTATGTTGTGTGGAATTGTGAGCGGATAACAATTTCCCCACTTCGATGCGTTTCATTTCG

### GC degradation profiles

GC degradation profiles were created to determine which component of Jet-A fuel is consumed by mutant strains as compared to the wild-type strain. The bioassays were performed as described by Striebich et al*.* (7). One-milliliter samples of *P. aeruginosa* ATCC 33988 wild-type cells and mutant cells were inoculated at 0.03 OD in a 10 mL glass vial, and 10 µL of Jet A aviation fuel was added. The samples were then incubated in a 28°C incubator for a period of 13 days without opening the vial. M9-fuel sample without bacteria was used as the control. After incubation, organic materials were extracted using 2 mL of high-performance liquid chromatography-grade hexanes (Fisher Scientific). The recovered extractants were analyzed by GC-MS (Agilent 7890 gas chromatograph and Agilent 5973 mass spectrometer). The final concentration of each compound in both the sample and negative control was initially normalized to farnesane (a non-degradable hydrocarbon by *P. aeruginosa*). The data were also normalized to the average value of the initial negative control samples to account for evaporation loss over the duration of the experiment.

### *alkB1* and *alkB2* competition study

*P. aeruginosa* ATCC 33988 and *alkB1 and alkB2* mutant cultures were prepared as described above. Cells were co-cultured in a 1:1 ratio at 0.03 OD in 50-mL polypropylene tubes with 10 mL of cells and 10 mL of Jet A aviation fuel. Samples were incubated at 28°C and shaken at 200 rpm. The growth of an individual mutant was assessed using a single set of PCR primers designed to only amplify the DNA sequence unique to the mutant, allowing them to selectively monitor its population size within the mixed culture (Table 2). Triplicate qPCRs were performed with the DNA using SYBR green master mix (Bio-Rad). Five microliters of the DNA was amplified using 0.2 µM of each mutant-specific primer. After the initial denaturation step at 95°C, samples were subjected to 40 cycles of PCR amplification using a 30-s annealing/extension step at 60°C.

**TABLE 2:** *alkB1* and *alkB2* mutant-specific primers

Target	Sequence name	Sequence
*alkB2* mutant	*alkB1 FWD*	ACT TCG TCT TCA CCA ACC TG
*alkB2* mutant	*alkB1 REV*	CTG CGG GCT ATC GTC ATA AT
*alkB1* mutant	*alkB2 FWD*	CACGAACTGATCCACAAGGA
*alkB1* mutant	*alkB2 REV*	ACCTTGAAGCCGGCATAG

### Statistical analysis

GraphPad Prism 6 software was used for statistical and nonlinear regression analysis. Data are presented as the mean ± standard deviation and curve fitting, and correlation coefficient (*R*^2^) was calculated. Statistical significance was determined when the *P*-value was <0.05.

## RESULTS

### Differentiation of bacteria/yeast/fungal spores from fuel samples

Our preliminary experiments were devoted to extracting microbes in jet fuels using liquid/liquid extraction methods and isolating microbes from hydrophobic hydrocarbons into more hydrophilic media/buffers before staining cells. Although some polar hydrocarbons were not fully removed from the aqueous phase, their presence did not interfere with the staining protocols. Fuel also contains a large number of auto-fluorescent particles, which were eliminated by setting the detection threshold for green and yellow fluorescence to obtain sufficient contrast between different cell types. The FCM assay has been demonstrated to accurately identify bacteria from yeast in the presence of hydrocarbons based on light scattering detectors and the use of green fluorescence (Fig. S1). When bacteria and yeast were inoculated to a fuel/minimal media mixture in different cell concentrations, the cell numbers were estimated using FCM and the traditional plate count method, and there was a strong correlation between the two methods. The observed detection limit of the FCM assay was 10^3^ and 10^2^ for bacteria and yeast, respectively (Fig. S2). However, concentrating and enriching the cells can be used to detect lower cell concentrations. Fungal spores are resting structures with a very limited metabolism, but SYTO 9 can stain nucleic acids in fungal spores to produce a detectable fluorescent emission. There is an extensive variability in spore staining by SYTO 9, yet FCM was able to detect a well-defined fungal spore population (Fig. S3). The fungal spore detection assay and inoculation of spores at different concentrations with the fuel/minimal media mixture indicate a good correlation between the FCM assay and the plate count methods with the detection limit of 10^2^ spores/mL. We observed that fungal spores are more concentrated near the fuel/minimal media interface than in the fuel or aqueous phase.

### *Pseudomonas aeruginosa* growth in jet fuel and differentiation of viable, injured, and dead cells using FCM assay

**Fig 1:**
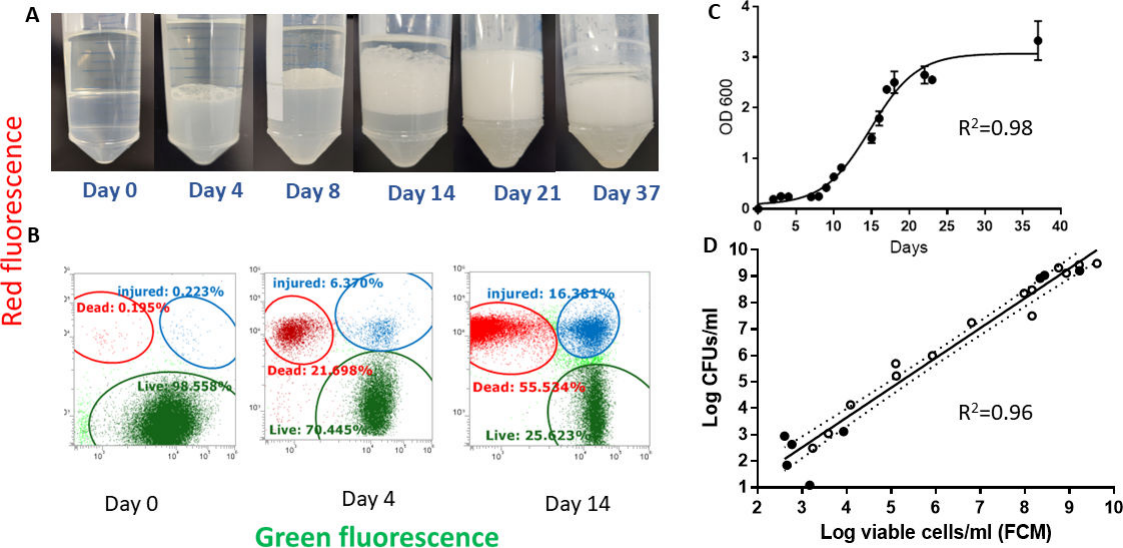
(**A**) *P. aeruginosa* growth in jet fuel (Jet-A); cells were grown in M9 minimal medium containing Jet A fuel. *P. aeruginosa* emulsify the hydrocarbons into the aqueous medium and increase the availability of hydrocarbons to microbial cells. (**B**) The viabilities of cell populations were determined by dual staining SYTO 9 with PI. The different colors define different *P. aeruginosa* populations in jet fuel. PI is excluded by viable cells with intact cell membranes, thus, the penetrating PI through the cell membrane indicates cell death or injured cells. Live cells are shown in green, and dead cells are shown in red. The blue populations are considered injured cells that are stained with both PI and SYTO 9. (**C**) The OD_600_ data shows *P. aeruginosa* grown in jet fuel has a typical sigmoidal growth with characteristic lag, exponential, and stationary phases. (**D**) The viable cells that were determined by the FCM assay correlate well with the plate count method. Solid circles indicate samples isolated from fuel or fuel/water interface, and open-hollow circles indicate samples isolated from the water phase. Assays of individual samples were performed in triplicate. The data point is calculated by taking the mean of three separate, independent biological replicates.

Although several viability markers are available, we found PI/SYTO 9 dual staining to be very reliable and the most straightforward method in detecting live and dead cells in the presence of hydrocarbons, and the technique has proven to work well to detect dead and live cells (Fig. 1). This study shows that *P. aeruginosa* growth in jet fuel (Jet A) can be accurately assessed using flow cytometry. The OD_600_ data showed that *P. aeruginosa* grown in jet fuel has a typical sigmoidal growth with characteristic lag, exponential, and stationary phases (Fig. 1C). In the exponential phase of the growth curve, bacteria produced significant amounts of biosurfactants at the fuel/water interface (Fig. 1A). *P. aeruginosa* are known to produce biosurfactants, such as rhamnolipids, which are amphiphilic compounds with surface-active properties. These biosurfactants can lower surface tension and facilitate interaction with hydrophobic fuel molecules. Our experiment shows the fuel layer was completely emulsified within 21 days after inoculation (Fig. 1A). When co-staining with PI and SYTO 9, it is only SYTO 9 that can enter through the cell wall regardless of their membrane integrity, and PI can only enter cells with compromised membranes, binding to DNA and RNA and emitting a red fluorescent signal. While it is correct to interpret that green fluorescence-emitting cells are “live” and red fluorescence-emitting cells are “dead,” there are a significant number of cells stained with both SYTO 9 and PI that are producing green and red signals. These are “injured” cells that allow penetration of a certain amount of PI along with SYTO 9. The flow cytometer can detect live, dead, and injured cells simultaneously from the fuel, fuel/water interface, and water phase. Although the fuel phase and fuel/water interface bacteria are extracted into the water phase before staining, a significant amount of fuel molecules are dissolved in the water phase. It is interesting to note that the fuel does not interfere with the staining protocol. As the growth progresses, *P. aeruginosa* accumulates a large number of dead cells and a significant number of injured cells (Fig. 1B). The FCM assay can detect all different cell types, such as live, dead, and injured cells in less than 30 min in a single sample, while plate counts detect only live and replicating cells after 48–72 hours of incubation. The viable cells that were determined by the FCM assay correlate well with the plate count method (Fig. 1D). The assay can be used to estimate cell viability in fuel, fuel/water interface, and water phase accurately.

### DiEGME induces viable nonculturable cells

**Fig 2:**
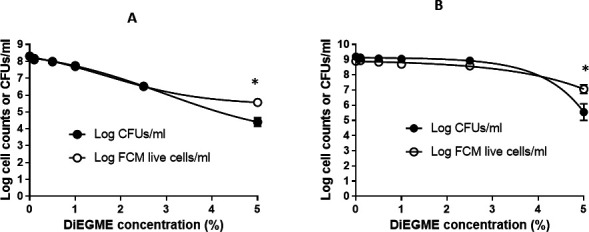
Dose response of *P. aeruginosa* to DiEGME measured by FCM compared with the plate count methods. Live cell populations were identified using live/dead staining of the sample, and the subsample was plated on TSA media and CFUs were determined. A starting cell concentration of 6 × 10^5^ cells/mL was used. (**A**) Twenty-four hours after DiEGME treatment, (**B**) 7 days after DiEGME treatment. Error bars represent a standard deviation of *n* = 3. * indicates the sample is significantly different (*P* < 0.05). The data point is calculated by taking the mean of three separate, independent biological replicates.

A fuel system icing inhibitor, DiEGME, has known antimicrobial properties, and this experiment shows that some cells of *P. aeruginosa* are viable but nonculturable when exposed to the fuel additive. DiEGME has both hydrophilic and hydrophobic functionalities and is soluble both in fuel and water phases, but there is much more efficient partitioning into the water phase (57). The fuel/water mixture was treated with varying concentrations of DiEGME, and the viability of cells was assessed using propidium iodide exclusion assay and plate count method after exposure to DiEGME in different time intervals over the course of the experiment (Fig. 2).

Our experiments demonstrated that 5% DiEGME with longer incubation (5 days) rendered *P. aeruginosa* cells nonculturable on TSA plates but showed plasma membrane integrity based on PI exclusion assay (Fig. 2). This indicates that DiEGME-treated cells were VBNC. In contrast, after 24 hours of incubation, the control cells were grown to a significantly high cell concentration of 2.07 × 10^8^ and 2.01 × 10^8^ cells/mL estimated by FCM and plate count method, respectively, and no significant VBNC cells were observed in the control study. Longer incubation (5 days) of 5% DiEGME-treated cells increased the cell number to 1.27 × 10^7^ based on FCM assay, but a significant number of these cells were unable to produce colonies on culture plates. The study indicates that 5% DiEGME is bacteriostatic and will prevent the growth of bacteria for at least the first 24 hours. We did not observe a significant reduction in starting inoculum after 24 hours based on PI exclusion assay measured by FCM, but 99.99% of cells detected by FCM failed to produce colonies on the culture plate. Day 7 analysis shows that the 5% DiEGME-exposed *P. aeruginosa* cells have undergone cell division, and a significantly higher number of cells were observed by FCM compared to day 1 exposure. However, the majority of these cells (98.07%) did not produce colonies on TSA culture plates.

### Expression of alkane monooxygenase genes (*alkB1* and *alkB2*)

Previously, we found that *alkB1* and *alkB2* gene promoters are inducible (8). The *alkB1* and *alkB2* promoters were individually fused to a GFP gene in pHERD20T vector and transformed to *alkB1* and *alkB2 P. aeruginosa* mutants, respectively. In this study, we used GFP-fused *alkB1* and *alkB2* promoters to detect *alkB1* and *alkB2* gene expression in the presence of jet fuel using flow cytometry. Based on the fluorescence of the *alkB-gfp* fusions, the experiments revealed that *alkB2* starts to express as little as 4 hours after cell exposure to Jet A fuel, while *alkB1* expresses at least 24 hours after activation of the *alkB2* promoter. Although *alkB2* is expressed in less than 4 hours after contact with alkanes, it is only 10% of *alkB2*-gfp cells that exhibit expression. This indicates heterogeneity in gene expression at a cellular level, and FCM was able to capture the dynamics of gene expression and metabolic status in mixed cell populations.

**Fig 3:**
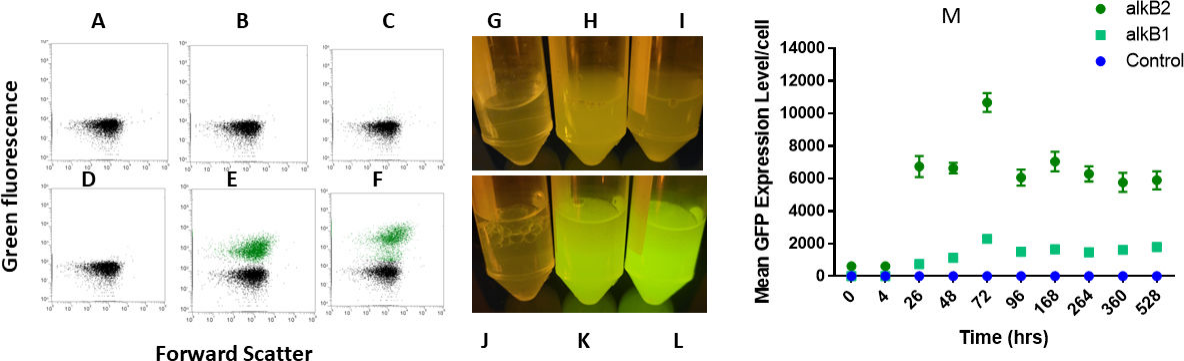
FCM dot plots of the GFP fluorescent intensities produced by *alkB1* and *alkB2* promoters. (**A**) Empty pHERD20T plasmid in *P. aeruginosa* in glycerol, (**B**) pHERD20T::*alkB1::gfp* reporter vector in *P. aeruginosa* in glycerol, (**C**) pHERD20T::*alkB2::gfp* reporter vector in *P. aeruginosa* in glycerol, (**D**) empty pHERD20T plasmid in *P. aeruginosa* in jet fuel, (**E**) pHERD20T::*alkB1::gfp* reporter vector in *P. aeruginosa* in jet fuel, (**F**) pHERD20T::*alkB2::gfp* reporter vector in *P. aeruginosa* in jet fuel, (**G**) test tubes containing *P. aeruginosa* carrying the empty plasmid in glycerol, (**H**) test tubes containing *P. aeruginosa* carrying the pHERD20T::*alkB1::gfp* reporter vector in glycerol, (**I**) test tubes containing *P. aeruginosa* carrying the pHERD20T::*alkB2::gfp* reporter vector in glycerol, (**J**) test tubes containing *P. aeruginosa* carrying the empty plasmid in fuel, (**K**) test tubes containing *P. aeruginosa* carrying the pHERD20T::*alkB1::gfp* reporter vector in jet fuel, (**L**) test tubes containing *P. aeruginosa* carrying the pHERD20T::*alkB2::gfp* reporter vector in jet fuel, (**M**) mean GFP fluorescent intensity over time for *alkB1* and *alkB2* promoter-induced expression compared to the empty plasmid. Error bars represent a standard deviation of *n* = 3. The data point is calculated by taking the mean of three separate, independent biological replicates.

Despite time differences in activation, both genes work together to degrade alkanes in jet fuel. The peak expression of both *alkB1* and *alkB2* was observed during the early log phase of growth approximately 72 hours after inoculation (Fig. 3). We noticed that regardless of growth phase, *alkB2* expression is significantly higher than *alkB1* expression. Both genes are expressed throughout the exponential and stationary growth phases and remained consistent with no clear transition between these two growth phases. In contrast, we noticed weak expression of *alkB1* and *alkB2* when cells grew in glycerol as the carbon source (Fig. 3). We demonstrate here that FCM is a good tool to measure temporal dynamics of gene expression in bacteria grown in hydrocarbons.

### The role of alkane monooxygenase-1 and alkane monooxygenase-2 in degradation

**Fig 4:**
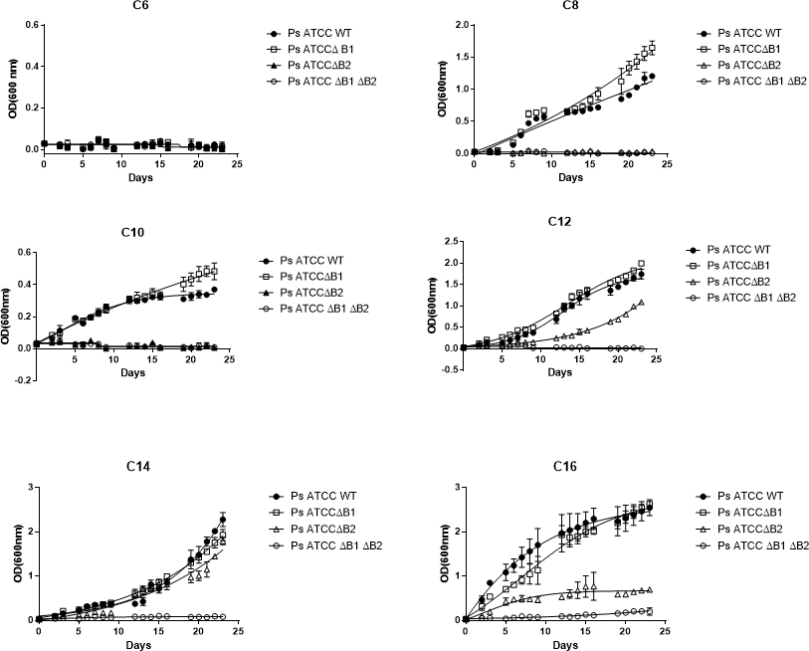
Substrate specificity of *P. aeruginosa* ATCC 33988 wild type and *alkB1, alkB2,* and *alkB1/alkB2* double mutant strains for individual normal alkanes as sole carbon sources. M9 minimal medium was supplemented with *n*-C6, *n*-C8, *n*-C10, *n*-C12, *n*-C14, and *n*-C16 as a carbon source, and the growth was measured using a spectrophotometer. Error bars represent a standard deviation of *n* = 3. The data point is calculated by taking the mean of three separate, independent biological replicates.

There are two alkane monooxygenases that exist in the *P. aeruginosa* ATCC 33988 genome (16), but their metabolic diversity has not been elucidated. In this study, we created *alkB1* and *alkB2* single mutants and an *alkB1/alkB2* double mutant and grew them in individual alkanes *n-*C6-*n*-C16. The substrate specificity of mutants was characterized using pure straight chain alkanes *n*-C6, *n*-C8, *n*-C10, *n*-C12, *n*-C14, and *n*-C16 as sole carbon sources. *P. aeruginosa* ATCC 33988 wild type and *alkB1* mutant were shown to degrade normal alkanes *n*-C8, *n*-C10, *n*-C12, *n*-C14, and *n*-C16, and there are instances where the *alkB1* mutant grew faster in *n-*C8 and *n-*C10 alkanes than the wild type (Fig. 4). We hypothesize that *alkB1* mutants continuously evolve in the hydrocarbon-rich environment to utilize certain normal alkanes efficiently. This indicates the alkane monooxygenase-2 gene has a broad substrate range compared to the *alkB1* (Fig. 4). However, the *alkB2* mutant did not grow at all in *n-*C8 or *n-*C10, indicating alkane monooxygenase-1 cannot utilize *n*-C8 or *n*-C10 as a carbon source. Also, the growth of the *alkB2* mutant in *n*-C16 was significantly attenuated compared to the wild type or the *alkB1* mutant. Genetically defective mutants lacking both alkane hydroxylase genes failed to degrade any of the normal alkanes (*n*-C6-*n*-C18) as a sole carbon source (Fig. 4). Finally, the growth characterization indicates that *P. aeruginosa* ATCC 33988 cannot degrade hexane (*n-*C6). The *alkB2* mutant degradation rate of *n*-C12 or *n-*C16 alkanes was slower compared to the *alkB1* mutant, which indicates that the AlkB2 monooxygenase has a higher activity compared to the AlkB1 monooxygenases. However, *alkB1* and *alkB2* mutants and wild-type strain grew essentially the same on *n*-C14 in the early log phase, but apparent variation was found in the late log phase, where the *alkB2* mutant grew slower on *n*-C14. This indicates that the collective action of these two enzymes degrades *n-*C14, and one enzyme can often be compensated by the other monooxygenase. The data also indicate that two alkane utilization pathways can coexist in *P. aeruginosa* ATCC 33988, and they are differentially expressed in response to *n*-C6*-n*-C16 *n*-alkanes found in jet fuel. Currently, the mechanism underlying this differential expression is not well understood.

### Selective consumption of n-alkanes by *P. aeruginosa* mutants and mutant competition study in co-culture experiments

**Fig 5:**
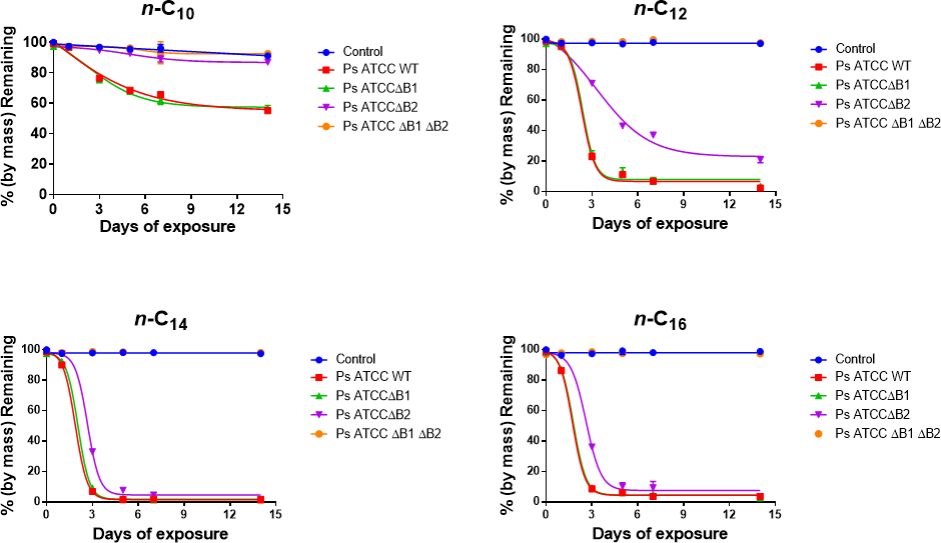
Selective consumption of Jet A fuel hydrocarbons by *P. aeruginosa* ATCC 33988 wild type and mutants. Degradation rate of normal alkanes based on molecular weight. The samples were analyzed by GC × GC with dual flame ionization detection and mass spectrometry detection. Error bars represent a standard deviation of *n* = 3. The data point is calculated by taking the mean of three separate, independent biological replicates. The final concentration of each compound, in both the sample and negative control, was initially normalized to farnesane (a non-degradable hydrocarbon by *P. aeruginosa*). The data were also normalized to the average value of the initial negative control samples to account for evaporation loss over the duration of the experiment.

**Fig 6:**
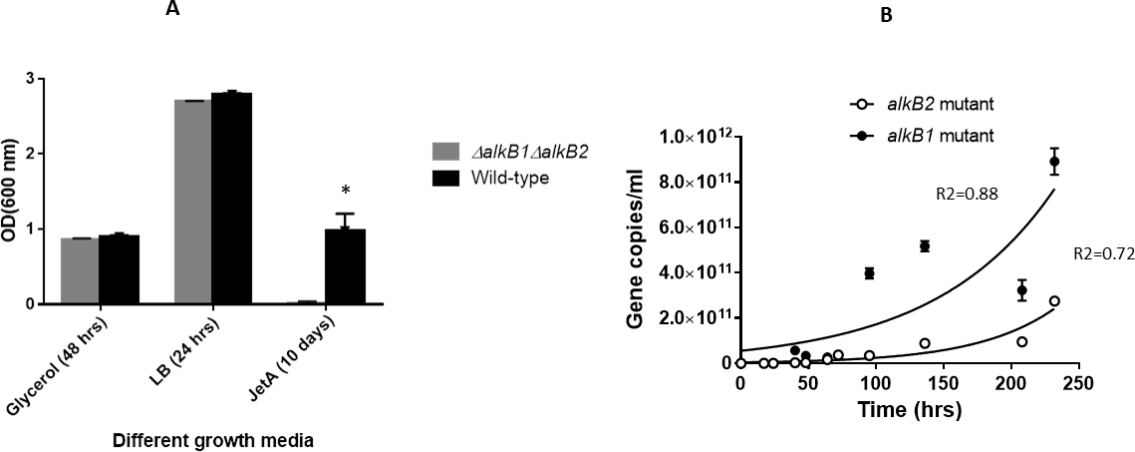
(A) Growth characteristics of the *alkB1* and *alkB2* double mutant strain in different growth media. Note: double mutant failed to grow in Jet-A fuel but grew well in M9 minimal media containing 0.2% glycerol or LB medium. (B) The *alkB1* and *alkB2* mutant competition study: co-cultured *alkB1* and *alkB2* mutants (mixed together in 1:1 ratio) in jet-A fuel, and growth was evaluated using PCR (using mutant-specific primers: Table 2). Error bars represent a standard deviation of *n* = 3. The data point is calculated by taking the mean of three separate, independent biological replicates

The substrate specificity and alkane monooxygenase activity of the two monooxygenases were further characterized using GC-MS on mutant strains (Fig. 5). Jet fuel composition analyses performed by two-dimensional gas chromatography (GC × GC) after mutants were grown in jet fuel provided new insights into the type of alkanes degraded by *P. aeruginosa* ATCC 33988 mutants. The GC results further confirmed the data presented in growth studies using individual normal alkane consumption analyses. The GC results showed that the *alkB2* mutant could not degrade *n-*C10, and the degradation of *n*-C12, *n*-C14, and *n*-C16 was slower in the *alkB2* mutant compared to the *alkB1* mutant (Fig. 5). The *alkB1* and *alkB2* double mutant could not degrade any of the normal alkanes tested (Fig. 5) and agreed well with the growth studies (Fig. 4). Furthermore, the double mutant failed to grow in Jet-A fuel but grew well in M9 minimal media containing 0.2% glycerol or LB medium (Fig. 6A). The *P. aeruginosa* ATCC 33988 *alkB1* and *alkB2* double mutant strain failed to degrade Jet-A fuel, suggesting that *P. aeruginosa* do not have mechanisms to degrade molecules, such as isoparaffins, cycloparaffins, and aromatics in Jet-A. When *alkB1* and *alkB2* mutants were co-cultured (mixed in 1:1 ratio), their fitness in jet fuel was evaluated using PCR (using mutant-specific primers). We found the *alkB1* mutant grew well and outcompeted the *alkB2* mutant (Fig. 6B), suggesting the *alkB2* pathway provides survival advantage and greater fitness for *P. aeruginosa* ATCC 33988 to grow and proliferate in alkanes.

## DISCUSSION

Biodegradation is one of the efficient ways of removing pollutants from the environment. Alkanes are energy-rich carbon molecules, and their utilization by microbes plays a key role in the carbon cycle. Alkane monooxygenase oxidizes a range of straight-chain alkanes of different carbon lengths and is widespread in the microbial community, including *P. aeruginosa* (62–67). AlkB2 monooxygenase has a distinct catalytic mechanism that dictates its substrate specificity (9). There is a demand and widespread interest in evaluating microbial community structures and their metabolic potential in hydrocarbon environments. Systematic detection and analysis of the dynamic behavior of microbial populations without affecting their niche are important to understand their activity and processes.

We have characterized alkane degradation pathways of *P. aeruginosa* ATCC33988 and compared the activity of two alkane monooxygenases (AlkB1 and AlkB2) on different alkanes. We concluded that AlkB2 is more effective than AlkB1 monooxygenase, and the *alkB2* pathway is induced earlier than *alkB1* in *P. aeruginosa* ATCC 33988 cells. These results agree with the findings reported in earlier research by Marín et al. (65), and it signifies the importance of differential and preferential expression of *alkB* genes in *P. aeruginosa*. The substrate specificity overlaps considerably and suggests that both enzymes act on the range of *n*-C12-*n-*C16 simultaneously. Overall, AlkB1 has a narrow substrate range and a weaker and late induction that summarizes its less significant role in hydrocarbon degradation in *P. aeruginosa* ATCC 33988. Besides the chain length of straight chain alkanes, their concentrations can also significantly influence the expression of *alk* genes involved in hydrocarbon degradation, as different levels of alkane exposure can trigger varying levels of gene activation in *P. aeruginosa*, leading to differential expression of the relevant genes, including regulatory mechanisms. However, having two alkane monooxygenase genes (*alkB1* and *alkB2*) and differential expression of *alkB1* and *alkB2* can be beneficial. For example, if one alkane monooxygenase becomes mutated and loses its function, the other alkane monooxygenase can still utilize alkanes. Having two alkane monooxygenases provides an important survival strategy as the two genes can coordinate gene expression in response to environmental changes. This allows them to adapt and survive in niches containing straight-chain alkanes with different carbon lengths.

The opportunistic human pathogen *P. aeruginosa* is biochemically versatile and one of the most important alkane-degrading bacteria in terrestrial environments. We confirmed previously that *P. aeruginosa* activates efflux pumps in the presence of hydrocarbons (8, 9, 12). Therefore, activation of efflux pumps in the presence of hydrocarbons can efflux important therapeutics, such as antibiotics, and export virulent determinants and contribute to bacterial pathogenesis. Therefore, prior exposure to hydrocarbons can result in a change in virulent factors and the host’s response. The degradation of straight-chain normal alkanes involves hydroxylation of the terminal methyl group to a primary alcohol, which is further oxidized to an aldehyde and fatty acids. Fatty acids are conjugated to co-enzyme A (CoA) and further oxidized via β-oxidation pathway to generate acetyl CoA. The *P. aeruginosa* ATCC 33988 genome encodes two active alkane monooxygenase genes *alkB1* and *alkB2* (16). Understanding the roles of two monooxygenases in metabolizing alkanes in *P. aeruginosa* ATCC 33988 is important to understand its metabolic processes in hydrocarbon environments. Our study suggests that two alkane monooxygenases of *P. aeruginosa* ATCC 33988 have preferential substrate specificity to provide broad-spectrum metabolism and metabolic flexibility to survive and proliferate in ecosystems containing hydrocarbons.

This study indicates that two alkane utilization pathways can coexist in *P. aeruginosa* ATCC 33988. However, the *alkB2* pathway appeared to be more critical for *P. aeruginosa* to survive and metabolize alkanes. Broader substrate specificity and, specifically, early induction make *alkB2* more important and provide *P. aeruginosa* ATCC 33988 a fitness advantage in the ecosystem. This is further confirmed when the *alkB1* mutant outcompeted the *alkB2* mutant in a co-culture experiment. Having two enzyme systems can provide a selection advantage in constantly changing environments for the success of *P. aeruginosa* ATCC 33988 to survive in the environment. In summary, we gained novel insight into *alkB1* and *alkB2* activity essential for alkane degradation in *P. aeruginosa* ATCC 33988. The mechanism that regulates *alkB* genes in *P. aeruginosa* ATCC 33988 is poorly understood, but recent work indicates that regulatory proteins, such as lcaR and GntR, play a role in the regulation of *alkB2* genes (68, 69). However, both *alkB1* and *alkB2* are responsible for degrading *n*-C12-*n*-C16, and these genes are not expressed in the presence of 0.2% glycerol, indicating there is tight control of gene expression of *alkB1* and *alkB2* achieved through the concerted action of transcription factors, such as regulatory proteins (23, 70), and small RNAs as shown for *P. putida* GpO1 (25).

GFP has been used extensively to visualize spatial and temporal forms of gene expression. Here, we systematically measured the temporal expression of two *alkB* genes in *P. aeruginosa* in the presence of jet fuel. By cloning the reporter *gfp* gene downstream of *alkB* promoters, *alkB* genes’ expression can be monitored in real time by measuring the green fluorescence emitted by GFP through flow cytometry. Our study indicated that the *alkB2* gene is induced preferentially during the early lag phase of growth, while *alkB1* is induced after induction of *alkB2*. With the knowledge that *P. aeruginosa* produce micro-colonies after exposure to fuel (9), it is advantageous to know gene expression in structured communities. Our results indicate that significant gene expression heterogeneity exists for *P. aeruginosa* in fuel cultures. The deletion of both *alkB1* and *alkB2* genes caused *P. aeruginosa* ATCC 33988 not to grow in jet fuel, indicating that it cannot utilize other hydrocarbons, such as aromatics, isoparaffins, and cycloparaffins as carbon sources. This study concluded that *P. aeruginosa* ATCC 33988 prefers to degrade straight alkanes over their isoparaffinic counterparts. Having two alkane degradation pathways with different substrate ranges is vital for *P. aeruginosa* ATCC33988 to survive in alkane-rich environments. This study also confirms that two soluble P450 alkane hydroxylases (PA2475 and PA3331) in *P. aeruginosa* ATCC 33988 are apparently not acting on *n*-C6-*n-*C18 normal alkanes present in jet fuel. Apparently, Cytochrome P450 alkane hydroxylases are common in alkane-degrading bacteria lacking membrane alkane hydroxylases (71).

Despite the fact that FCM-based methods have been increasingly used to detect microbes across many fields, such as food microbiology (36–41), water microbiology (42, 43), and clinical microbiology (44), this methodology has not been used to evaluate the contaminants in fuel tanks or study the hydrocarbon decontamination process in the environment. This is because hydrocarbons are hydrophobic, and their non-polar nature makes it difficult to run directly through the FCM. Here, we show that FCM is a powerful method allowing detection and enumeration of different microbial populations in hydrocarbon-containing jet fuel. The work reported herein is a first step in applying flow cytometry for rapid monitoring of microbial communities in hydrocarbons that are recalcitrant in nature and have high hydrophobicity, making it difficult for microbes to proliferate and degrade. For bioremediation, developing microbial strains with fast growth rates and good fuel degradation capabilities is essential. We assume that fast-growing strains need to be evaluated for their growth, viability, and vitality *in vitro* in the presence of toxic waste before they are released into the environment, and the assays developed here can be used effectively for this purpose. The live/dead/injured assay that has been tested in this study provides a wealth of information to monitor microbial performance and pursue strains that could survive and grow faster for other biotechnology applications. Our results indicate that hydrocarbon stress promotes population heterogeneity. In this case, heterogeneity exists in genetically identical populations of *P. aeruginosa* after exposure to hydrocarbons. Linking functional fluorescent dyes with fast cell detection and differentiating capabilities with FCM reveals additional information that makes it beneficial for the study of mixed heterogeneous populations that have not been appreciated by other methodologies. Heterogeneity and cell-to-cell difference are driven by a diverse set of factors, including genetic factors. Our previous research shows that exposure to hydrocarbon-rich jet fuel forms cell aggregates (9), which create a unique microenvironment and cellular interactions, where the outermost cell layers are directly exposed to hydrocarbons, while inner cells are less exposed. Currently, there has been a growing interest in the mechanisms that create heterogeneity within a clonal population where environmental stresses and adaptation are put forward as predisposing factors for such cell-to-cell variation within a clonal population (2). A subset of genes is regulated by quorum sensing in *P. aeruginosa,* and these diffusible signaling molecules (autoinducers) may not be evenly distributed in microenvironments of cell aggregates. Cell scale heterogeneity and population scale graded responses to continuous environmental variation have been observed when *P. aeruginosa* was exposed to a different carbon environment (72). In response to environmental changes from a rich LB medium to a hydrocarbon-rich fuel environment, each bacterial cell may have responded differently, and as a result, they may have produced distinct phenotypic groups. Dodson et al. (73) observed distinct morphologies of cell subpopulations in a biofilm environment. Injured cells identified by FCM are considered as partially compromised cell membranes as they allowed some PI molecules to penetrate through the phospholipid membrane. Hydrocarbons are known to accumulate in the lipid bilayer and modify membrane composition, affecting the structural and functional properties of these membranes (74). Therefore, targeting cell membrane integrity is a good marker to assess the viability of microorganisms, particularly microbes growing in the presence of hydrocarbons. Therefore, cell scale heterogeneity leads to different responses when exposed to hydrocarbons. While some cells survive and proliferate, other cells are susceptible to toxic solvents.

## Data Availability

The genome sequence of *P. aeruginosa* ATCC 33988 has been deposited in NCBI GenBank under accession number JPQQ00000000 (16). The Pseudomonas Genome Database (https://www.pseudomonas.com/) provides *P. aeruginosa* PAO1 genome annotation and genome analysis data. The data that support the findings of this study are available upon request from the corresponding author.

## SUPPLEMENTAL MATERIAL

Supplemental figures jb.00508-24-s0001.pdf Figures S1 to S3.

Supplemental figure legends jb.00508-24-s0002.pdf Legends for Figures S1 to S3.

ASM does not own the copyrights to Supplemental Material that may be linked to, or accessed through, an article. The authors have granted ASM a non-exclusive, world-wide license to publish the Supplemental Material files. Please contact the corresponding author directly for reuse.
