# Supplementary figures and images for: Metabolic characterization of alkane monooxygenases and the growth phenotypes of Pseudomonas aeruginosa ATCC 33988 on hydrocarbons
Source: J Bacteriol. 2025 Mar 11;207(4):e00508-24. doi: 10.1128/jb.00508-24 (PMC12004949; doi:10.1128/jb.00508-24)

Figure S1

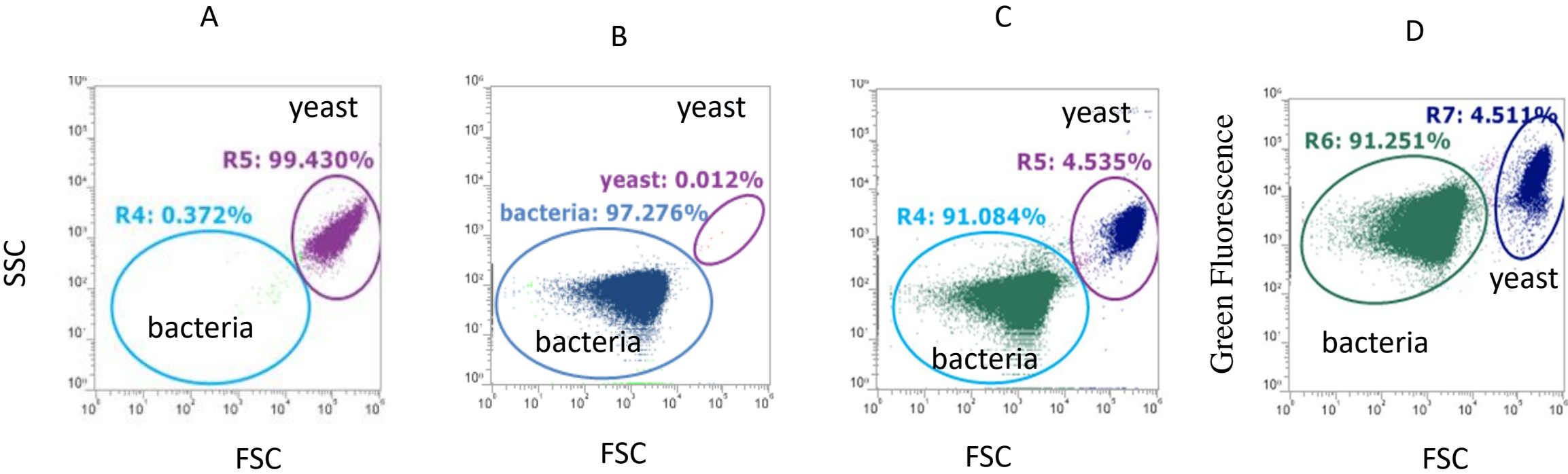

Figure S2

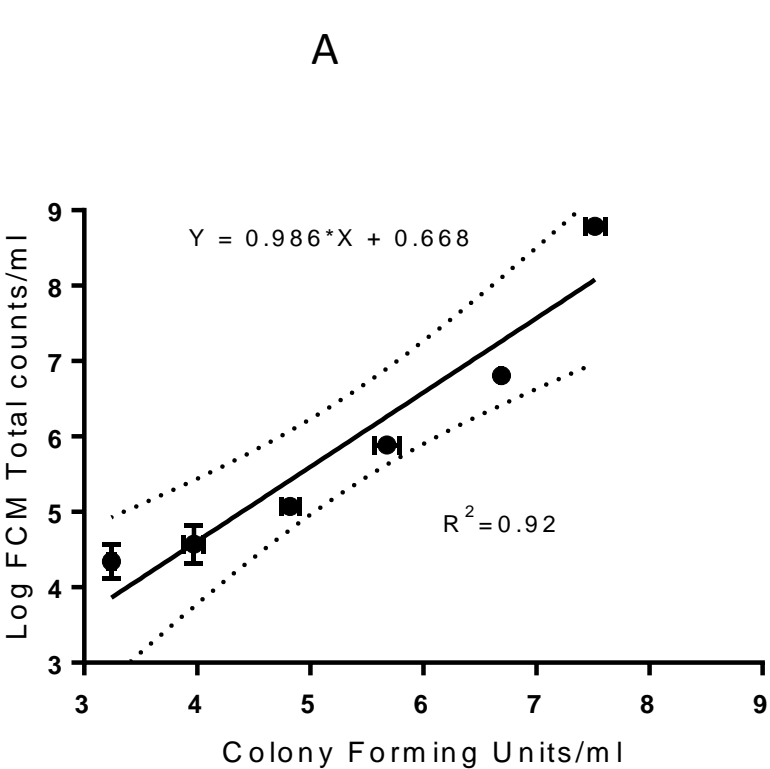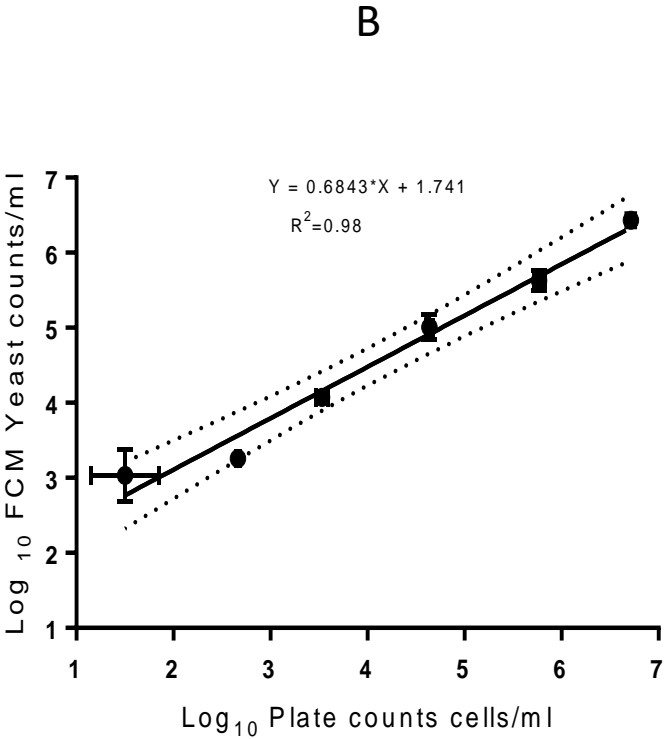

Figure S3

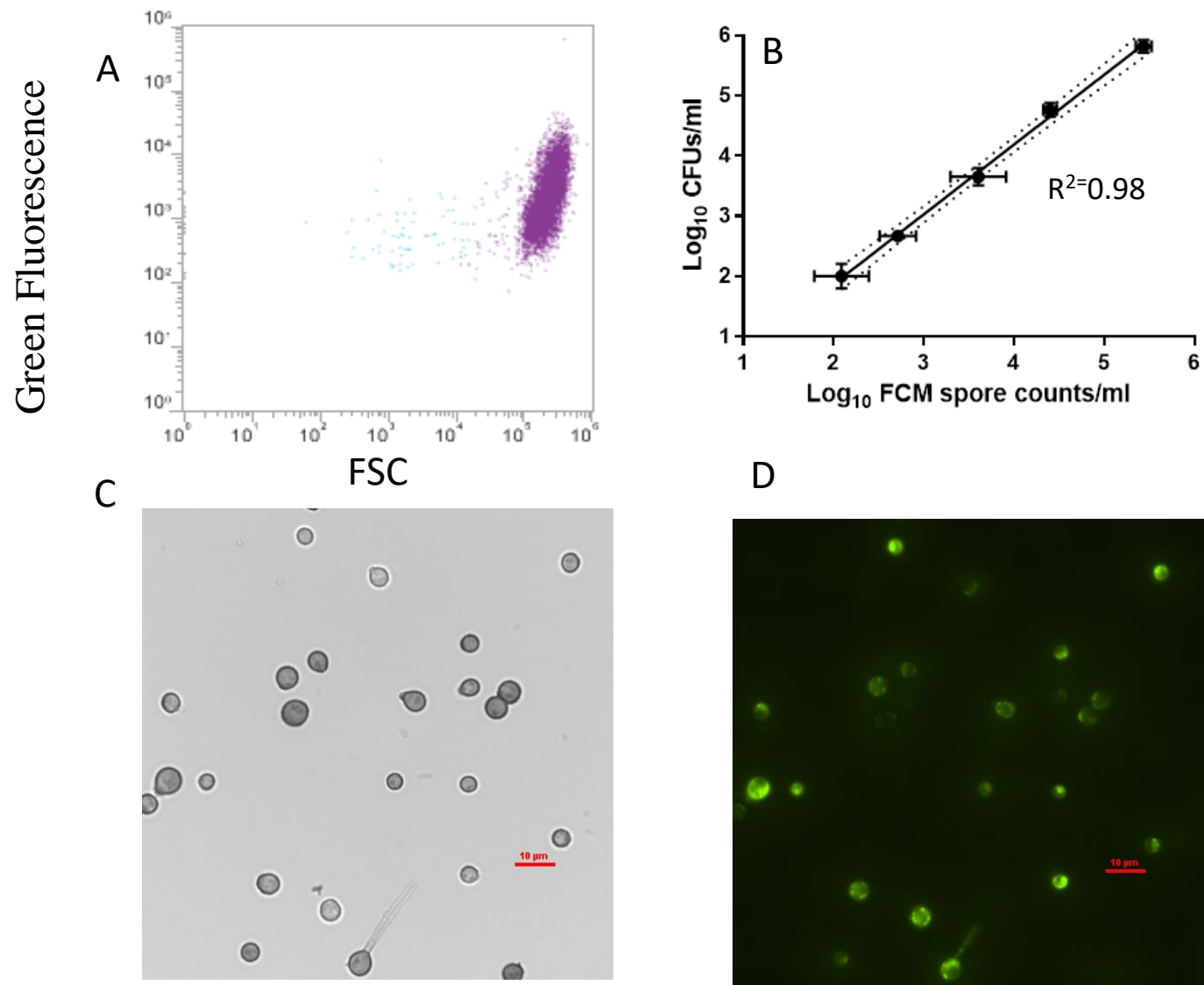

Supplement: Supplemental figures — Figures S1 to S3. [file jb.00508-24-s0001.pdf]
