# Supplementary material for: Metabolic characterization of alkane monooxygenases and the growth phenotypes of Pseudomonas aeruginosa ATCC 33988 on hydrocarbons
Source: J Bacteriol. 2025 Mar 11;207(4):e00508-24. doi: 10.1128/jb.00508-24 (PMC12004949; doi:10.1128/jb.00508-24)
Supplement: Supplemental figure legends — Legends for Figures S1 to S3. [file jb.00508-24-s0002.pdf]

Supplementary Figures

Figure S1. The assay can differentiate bacteria and yeast from non-cell background events using FCM dot-plots based on forward scatter (FSC) and side-scatter (SSC) (**A)** Yeast only (**B**) Bacteria only (**C)** Differentiation of bacteria and yeast in minimal media based on the side scatter and forward scatter (**D)** Bacteria and yeast differentiation based on the green fluorescence and forward scatter. Gating is an important feature in FCM to separate different cell types or cells from non-cell events. This experiment confirms distinct gated yeast, and bacteria populations allow for enumerations of bacteria and yeast separately from fuel, fuel water interface and water phase. The detection threshold was adjusted with green fluorescence to eliminate autofluorescence particles.

Figure S2. The detection limit and the correlation between FCM cell counts with Plate Count Method (**A)** bacteria (**B)** yeast. Viable bacteria and yeast cells were spiked at different cell concentrations and evaluated cell density using FCM and plate count methods and sub-samples were plated on TSA and determined CFUs/ml. Error bars represent a standard deviation of n=3.

Figure S3. FCM detection of fungal spores in water contaminated fuel samples. (**A)** Fuel samples were inoculated with different spore concentrations and samples were analyzed on the Attune NxT flow cytometer and sub-samples were plated on YPD and determined CFUs/ml. (**B**) Correlation between fungal spore count obtained from FCM and standard plate count methods. (**C)** Light microscopy image of fungal spores. (**D)** Fungal spores staining with SYTO 9. Error bars represent a standard deviation of n=3.
